# Diabetic Peripheral Neuropathy and Depression: Dancing with Wolves? - Mini-Review and Commentary on Alghafri *et al*. “Screening for depressive symptoms amongst patients with diabetic peripheral neuropathy”

**DOI:** 10.1900/RDS.2021.17.17

**Published:** 2021-08-01

**Authors:** Prashanth R.J. Vas, Nikolaos Papanas

**Affiliations:** 1 Diabetes Foot Clinic, King’s College Hospital, London, UK,; 2 King’s Health Partners’ Institute of Diabetes, Endocrinology and Obesity, London, UK,; 3 Diabetes Centre, Diabetic Foot Clinic, Second Department of Internal Medicine, Democritus, University of Thrace, Alexandroupolis, Greece.

**Keywords:** comorbidities, complications, depression, diabetes mellitus, diabetic neuropathy

## Abstract

The co-existence of diabetic peripheral neuropathy (DPN) and depression in subjects with diabetes is being increasingly recognized. The interaction of these two serious comorbidities may increase morbidity and mortality. An emerging thought is that persisting depression, along with stroke and cognitive dysfunction, may represent a cluster of potential microvascular injuries affecting the brain, which shares a common risk factor with DPN. Current evidence highlights metabolic and clinical covariates, which may interact in subjects with DPN and depression. However, there is a lack of rigorous enquiry into the confounding effect of cognitive dysfunction and vascular brain disease. Furthermore, high-quality longitudinal studies exploring the direct impact of these comorbidities on diabetes course and on the progression of the comorbidities themselves are lacking. Improved insights into comorbid DPN and depression may help to improve screening for and treatment of both these conditions.

## 1 Introduction

The association between diabetes mellitus and depression has long been recognized, particularly in type 2 diabetes (T2D). A diagnosis of T2D may increase the risk of incident clinical depression by approximately 25-52%, after adjusting for covariates and comorbidities [1, 2]. Indeed, a recent meta-analysis of epidemiological studies concluded that subjects with T2D had a 2-fold increased risk of major depressive disorder [3]. Conversely, individuals with depression exhibited an up to 1.5 times increased risk of developing T2D [4]. Likewise, depression in type 1 diabetes (T1D) is 3 times more prevalent than in the general population [5], with adolescents and younger adults with T1D tending to have disproportionately higher risk [6].

Importantly, comorbid diabetes and depression have been confirmed to increase the risk of mortality [7, 8].

In T2D, depression is linked with poor glycemic control and clinically significant micro- and macrovascular disease [8, 9]. In T1D, depression is associated with poor treatment adherence, higher diabetes distress scores [10], suboptimal glycemic control, and recurrent diabetic ketoacidosis [11, 12]. Diabetes-specific risk factors, such as micro- and macrovascular complications [13, 14], act in concert with traditional risk factors for depression, including female gender, lower levels of education, and psychosocial factors (e.g. childhood trauma and social deprivation), thereby creating a complex web of interactions [15].

## 2 Diabetic peripheral neuropathy and depression

Diabetic peripheral neuropathy (DPN) is a common complication of diabetes, affecting up to 50% of subjects with diabetes over their lifetime [16, 17]. Older age, longer diabetes duration, poor glycemic control, height, male gender, hypertension, dyslipidemia, and certain ethnicities represent the major risk factors for DPN [16, 17]. Sensory-predominant, length-dependent distal symmetrical sensorimotor neuropathy is the most common presentation, with an insidious onset and gradual clinical progression [18]. DPN can negatively impact on physical function secondary to neuropathic pain, Charcot osteoarthropathy, unsteadiness, and lack of postural control [18, 19]. However, when severe DPN with loss of protective sensation (LOPS) develops, the foot becomes particularly vulnerable to ulcerations, and amputations are its ultimate risk [16-19].

Given the high prevalence of both depression and DPN in combination, it is not surprising that these two comorbidities are closely associated. Clinical measures of DPN, such as the neuropathy disability score (NDS) and the vibration perception threshold (VPT) have been shown to be independently associated with the Hospital Anxiety and Depression Scale (HADS), a 7-item scale measuring the absence of positive affect and pleasure [19]. Recently, an association between another depression screening instrument, the Patient Health Questionnaire 9 (PHQ-9), and sudomotor dysfunction has been reported [20]. Symptoms of DPN, including reduced foot sensation, pain, and unsteadiness, are also related to depression, with more severe symptoms being associated with more marked depression scores [5, 19]. These observations have been confirmed in a recent large meta-analysis demonstrating that depression was independently associated with DPN (p = 0.002) with a moderate effect size (r = 0.28) [21].

## 3 The impact of the study by Alghafri *et al*.

The study by Alghafri et al. is of particular interest in the context of an association between DPN and depression [22]. Subjects with diabetes were examined for DPN and completed the PHQ-9 depression screening questionnaire. Diagnosis of DPN was based on abnormal 10g monofilament test or 128 Hz tuning test [22]. Subjects with DPN exhibited significantly higher mean PHQ-9 scores than those without DPN (6.09 ± 4.80 vs. 2.24 ± 2.63, p < 0.0001) [22]. Furthermore, a greater proportion of subjects with DPN had higher severity of depression (PHQ-9 ≥ 10) than those without DPN (26.6% vs. 2.0%) [22]. Interestingly, subjects with a history of diabetic foot ulcer (DFU) or peripheral arterial disease were excluded, which is an important strength of the study [22]. This new study adds to the growing appreciation that DPN and depression are closely associated.

Nevertheless, there are a few important limitations in the study by Alghafri *et al*. [22]. Firstly, the study sample was very small. Secondly, as duly acknowledged by the authors, the diagnostic criteria for DPN, in particular the 10g monofilament, are suitable to detect advanced DPN with LOPS, but are not sensitive enough for milder, incipient DPN [22]. To detect the latter reliably, clinical examination scores (notably the Neuropathy Disability Score or the Toronto Clinical Neuropathy Score) would have been more suitable [16, 18]. Admittedly, there is a lack of uniformity in the diagnostic criteria used for DPN in the studies evaluating both DPN and depression, making it difficult to compare them. Thirdly, the mean age of participants was 77 years [22]. While the diagnostic accuracy of PHQ-9 in the elderly has been confirmed, measures of DPN such as those used in the study by Alghafri et al. are intrinsically and inversely related to age and, therefore, less reliable in such old populations [16, 18]. Finally, the authors did not adjust for confounding factors, especially co-existent neuropathic pain [23].

A recent systematic review of comorbid DPN and depression identified a triad of clinical characteristics in such individuals: Older age (often >65 years of age), frailty (often with neuropathic pain and other diabetes complications), and poor glycemic control despite insulin treatment [24]. The presence of depression does not simply increase the risk of incident neuropathic DFU by two-fold [25, 26], it even negatively impacts the clinical outcome and may increase mortality [27]. An important message from the study by Alghafri et al. is that it reinforces the clinical need to screen for depression in subjects with LOPS [22]. This may also have economic ramifications. Indeed, it is estimated that subjects with DPN and depression have 50% higher healthcare costs than those with DPN only [28].

## 4 Association of cognitive dysfunction and vascular brain disease

One emerging concept is the recognition that depression, stroke, and cognitive dysfunction may be representations of cerebral microvascular disease [29]. Pivotal risk factors for the latter include hyperglycemia, insulin resistance, dyslipidemia, and hypertension [29], which are also the main risk factors for DPN [16-18]. Indeed, cognitive dysfunction is commonly present in subjects with DFU [30], who, in turn, frequently have DPN. Therefore, a major limitation in the current literature on comorbid DPN and depression is the lack of information on cognitive dysfunction and vascular brain disease.

## 5 Clinical practice for diabetic peripheral neuropathy and depression

At present, there are no specific screening or treatment recommendations for depression in DPN. In a recommendation paper focusing on primary care physicians, the American Diabetes Association has emphasized that all subjects with diabetes and DPN should be regularly screened for depression [31]. This is in accordance with evidence from interventional trials that treating depression may lead to simultaneous improvement in both diabetes and depression [32]. Nonetheless, we certainly need to know more about the clinical and biochemical characteristics as well as the sociodemographic factors in comorbid DPN and depression. New information should come from large and adequately powered prospective cohort studies. Last but not least, we need a consensus on the diagnostic criteria of DPN and depression to interpret reliably and compare future interventional trials.

## 6 Inhibition of RAS in diabetic nephropathy

In conclusion, DPN may co-exist with depression and vice versa. It is evident that the two conditions share common risk factors, mainly those classically associated with cardiovascular disease in diabetes. However, they also share certain clinical characteristics, in particular older age, frailty, neuropathic pain, and diabetes complications. While this constellation may be considered the equivalent of “birds of the same feather flocking together” [33], its clinical impact can be as fierce as a wolf pack. In everyday practice, subjects with LOPS should be evaluated for depression early, with the aim of avoiding resultant impoverishment in the quality of life [17, 25, 27, 34].
